# *In Vivo* Near-Infrared Fluorescence Imaging of Aqueous Humor Outflow Structures

**DOI:** 10.1155/2016/8706564

**Published:** 2016-05-24

**Authors:** L. Zeppa, L. Ambrosone, G. Guerra, M. Fortunato, C. Costagliola

**Affiliations:** ^1^Department of Ophthalmology, G. Moscati Hospital, 83100 Avellino, Italy; ^2^Department of Bioscience and Territory, University of Molise, Pesche, 86090 Isernia, Italy; ^3^Department of Medicine and Health Sciences, University of Molise, 86100 Campobasso, Italy; ^4^Department of Ophthalmology, Bambino Gesù Hospital, 00146 Rome, Italy; ^5^IRCCS Neuromed, Pozzilli, 86077 Isernia, Italy

## Abstract

The aim of this study has been to visualize the aqueous outflow system in patients affected by primary open angle glaucoma. A solution of indocyanine green (ICG) plus high viscosity viscoelastic solution was injected into the Schlemm canal during surgery in 10 glaucomatous patients undergoing canaloplasty. Soon after injection of the dye the borders of the scleral flap were completely stained due to partial reflux caused by the intrachannel resistance; progression of the dye along the Schlemm canal starting from the site of injection was then visualized. The filling of the collector channels was observed only in the patent portions of the Schlemm canal. The only noticeable aqueous veins were located in correspondence of the quadrant in which both the Schlemm canal and the collectors were patent. Lastly, a retrograde filling, of glomerular-shaped structures, deepest to the Schlemm canal was observed in the quadrants where the pathway was functioning. Our findings show that injection of a mixture composed of ICG and viscoelastic solution into the Schlemm canal allows a clear visualization of the functioning portions of the conventional outflow pathway. In addition, a retrograde filling of structures presumably located into the iris was also recorded.* Clinical Trial Registration*. Our study is registered in ISRCTN registry, number 54005880, DOI 10.1186/ISRCTN54005880.

## 1. Introduction

Eponymous “glaucoma” recognizes a group of ocular conditions characterized by progressive optic nerve damage associated with loss of visual function and, frequently, with elevated intraocular pressure (IOP). IOP increase is due either to a very infrequent rise of aqueous humor (AH) production or to a more frequent impairment of AH outflow. The balance between the rate of AH production (inflow) and the resistance that it encounters in its egress away from the eye (outflow) generates the level of IOP; however, in normal subjects equilibrium exists between aqueous humor production and drainage. Aqueous humor leaves the eye through two main pathways: one sensitive to eye pressure,* the conventional route* (trabecular meshwork, Schlemm's canal (SC), collector channels (CCs), aqueous veins (AV), and episcleral veins (EV)), and the other independent of eye pressure,* the nonconventional route* (uveal meshwork, ciliary muscle, suprachoroidal space, and sclera) [1]. In human, about 80%–90% of aqueous outflow takes place through the conventional outflow, whereas about 10%–20% passes through unconventional outflow. The major function of the conventional outflow pathway is to maintain the intraocular pressure within the critical range by modulating the outflow resistance of the aqueous humor. Structural modifications in this pathway can lead to raised intraocular pressure, greatly increasing the risk for developing glaucoma [2].

Despite the importance of verifying the healthy functioning of this system, especially in light of its implication in both medical and surgical approaches, no defined technique exists to visualize* in vivo* the structure responsible for the AH outflow. A wide range of imaging techniques to visualize the aqueous outflow system have been developed. Wessely [3], followed by Schulte [4], Goldmann [5], and Benedikt [6], described the aqueous pathway by injecting fluorescein directly into the anterior chamber (AC). The fluorescein, injected into the AC, becomes visible in ultraviolet light making the recording of aqueous humor outflow by photography possible. The drawbacks of this technique are due to the large volumes and high concentration of the fluorescein employed, to the delayed filling of the episcleral venous network, and to the leakage from scleral vessels. More recently, Grieshaber et al. [7] proposed a new approach by using a flexible microcatheter through which fluorescein is injected. This technique, called channelography, permits videorecording and evaluation of the aqueous outflow pathway. Limitations of the method consist of the immediate dye diffusion into channel network and the uveoscleral pathway. Noninvasive methods to display the outflow pathway from SC to the superficial vasculature comprise high frequency ultrasound (UBM 50 MHz) [8], time domain optical coherence tomography (OCT) [9, 10], and, more recently, swept source Fourier domain (SD) OCT systems [11]. The limitations of this method are the following: (1) it does not allow the evaluation of all the structures involved in the AH outflow pathway; (2) it does not allow both distinguishing arteries from veins and giving a quantitative comparison of vessel number and vessel diameter; (3) SD-OCT images are not real, being rather a 3D virtual casting of the aqueous outflow vasculature; thus OCT anatomical images do not reveal the functional status; and, lastly, (4) the* in vivo* 3D imaging acquisition is disturbed by eye movements [9, 10].

The purpose of the present study is to assess the aqueous outflow system in patients affected by primary open angle glaucoma (POAG) undergoing canaloplasty, using a flexible microcatheter filled with indocyanine green (ICG) diluted in viscoelastic solution. We thought that ICG, being a larger molecule than fluorescein and coupled to a viscous carrier, might be less prone to leak from the SC, therefore better visualizing the outflow pathway. Preliminary results of this technique have been already reported elsewhere as photoessay [12].

## 2. Methods

Surgical procedures were performed at the “S. Giuseppe Moscati” Hospital, Avellino, Italy, by one surgeon (L. Zeppa). Ten patients affected by POAG undergoing canaloplasty (four males and six females, mean ± SD ages of 51 ± 8 and 47 ± 9 years, resp.) were included in this pilot study. All patients were under maximum tolerated medical therapy and had been followed up at least 60 months before surgery. Exclusion criteria were narrow or closed iridocorneal angle, evidence of any secondary glaucoma, pigmentary dispersion, pseudoexfoliation, history of trauma, history of uveitis, any type of corneal disease, or previous refractive surgery. Informed consent was obtained from each patient after a detailed description of the procedure used and of the aim-work. The study was approved by the local institutional ethics committees and followed the tenets of the Declaration of Helsinki.

The study was registered in ISRCTN registry, number 54005880, DOI 10.1186/ISRCTN54005880.

### 2.1. Indocyanine Green (ICG) Tracer

The preparation of the ICG tracer was done under sterile conditions. A solution composed of ICG (ICG-PULSION 25 mg/50 mg, PULSION Medical Systems SE, Germany) and commercially available viscoelastic solution with high viscosity and high density (DisCoVisc, Alcon Laboratories Inc., USA) and with a final ICG concentration of 25 *μ*g/mL was used. The cartridge of the viscoinjector was filled with such mixed solution and attached to the microcatheter.

**Figure 1:**
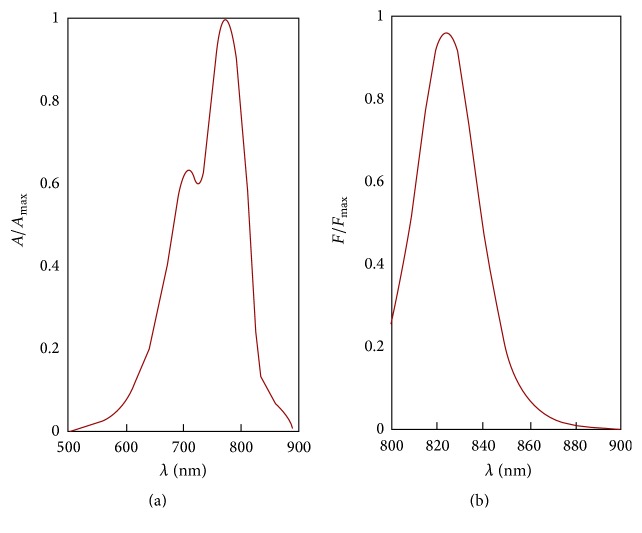
(a) UV-Vis spectrum and (b) fluorescence spectrum of aqueous solution of ICG 25 *μ*M at 30°C.

The commercial viscoelastic solution is a mixture formed by 1.6% of hyaluronic acid and 4% of chondroitin sulfate, with zero-shear viscosity of 75 mPa·s. Arshinoff and Jafari proved that the zero-shear viscosity correlates well with cohesiveness [13]; therefore, the viscoelastic solution soon dissipates into the canal after injection. ICG is a negatively charged tricarbocyanine dye with strong absorbing properties in the near-infrared range and is only weakly fluorescent in its unbound state. The UV-Vis spectrum of aqueous solution 25 *μ*M is displayed in Figure 1(a), whereas Figure 1(b) shows its fluorescence spectrum. The excitation and emissions wavelengths utilized for this spectrum were 778 nm and 810 nm, respectively.

### 2.2. Microcatheter

The microcatheter used (iTrack*™* 250A, iScience Interventional, Menlo Park, CA, USA) consists of 3 parts: an optical fiber which allows illumination of the distal tip for transscleral visualization, a support wire to provide pushing ability during catheterization, and a lumen through which fluids can be delivered. The overall diameter of the shaft of the microcatheter is 200 *μ*m with a blunt distal tip (250 *μ*m) to minimize potential tissue damage. A screw-driven syringe is connected to the proximal end of the microcatheter and delivers a precise volume of solution. One-eighth (1/8) of a turn on injector knob equals 150 *μ*L volume fluid injected.

### 2.3. Microscope

Visualization of the tracer in the outflow pathway was accomplished using the microscope OPMI PENTERO 900 (Carl Zeiss Meditec, Germany) taking advantage of the fluorescence application FLOW® 800 which allows the visual analysis of vascular blood flow dynamics in the range of infrared wavelengths (~800 nm).

### 2.4. Canaloplasty

Preliminary dissection of superficial and deep scleral flaps and subsequent unroofing of the SC were performed. Both ostia of the SC were dissected forward to the Descemet membrane to expose the trabeculo-Descemet window and facilitated the insertion of the microcatheter into the SC. The microcatheter was positioned in plane and in line to the SC and advanced into complete circumference while the location of the red blinking tip was observed through the sclera. During advancement of the tip, a fixed volume of ICG tracer (150 *μ*L) was gently injected through the microcatheter by 1/8 of a turn on the viscoinjector.

## 3. Results

**Figure 2:**
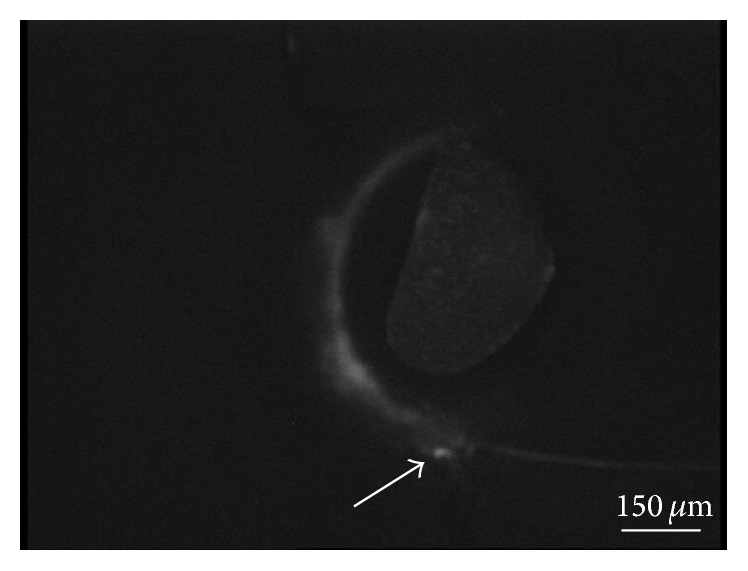
Progression of the dye along Schlemm's canal starting from the site of injection (arrow). The central shadow is due to the presence of the IR shell protection.

Soon after injection of the dye, there was impregnation of the borders of the scleral flap due to partial reflux caused by the intrachannel resistance; progression of the dye along the SC starting from the site of injection was then visualized (Figure 2).

**Figure 3:**
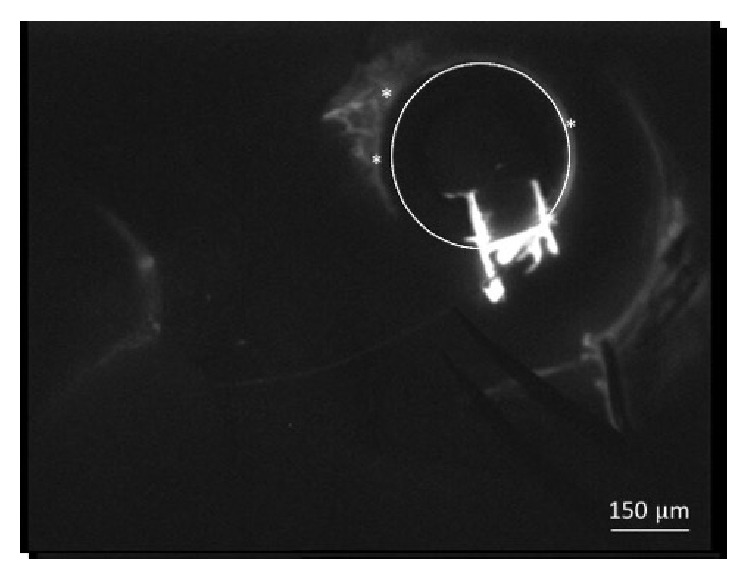
Filling of the collector channels (*∗*) observed in correspondence of the patent portions of Schlemm's canal whose boundary was indicated by a circle. The bright H shape is due to the impregnation of the borders of the scleral flap due to partial reflux caused by the intrachannel resistance to dye progression.

**Figure 4:**
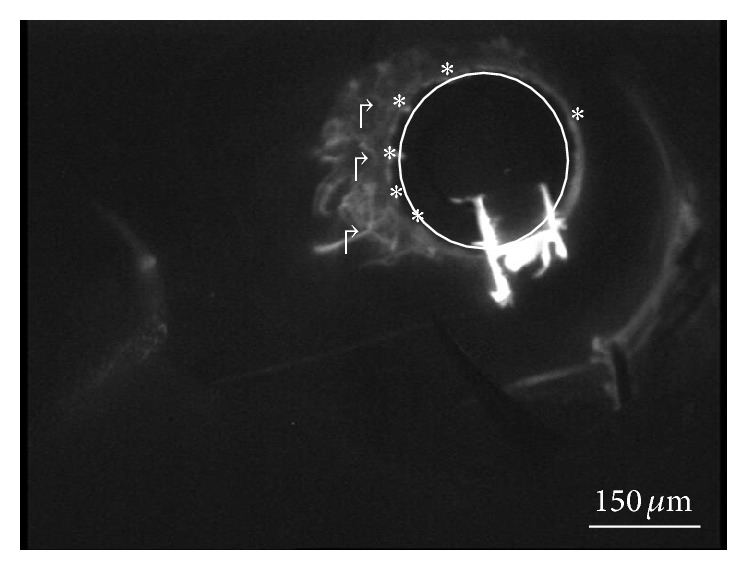
Filling of aqueous vein (↱) located in correspondence of the quadrant in which both Schlemm's canal whose boundary was indicated by circle and the collectors (*∗*) were patent.

In some eyes, the filling proceeded along 360 degrees whereas in others only a portion of the canal was visualized. In 3 patients, the percentage of filling was 70%, whereas in the remaining 7 patients this percentage decreased up to 50%. A correlation between SC filling and number of CCs visualized was observed. It is possible that an obstruction of the canal was present in such eyes. The filling of the collector channels occurred only in correspondence of the patent portions of the SC (Figure 3). However, even in presence of a patent SC over 360 degrees, the filling of the collector channels was not simultaneously visualized in all four quadrants. Furthermore, the collector CCs closer to the site of injection did not necessarily fill up before the farther ones. On the contrary, the filling seemed to be influenced by the resistance encountered by the dye to progress; in fact, after increasing the volume/pressure of infusion, visualization of some CCs, initially not perfused, occurred. The only noticeable aqueous veins were located in correspondence of the quadrants in which both the SC and the CCs were patent (Figure 4). The upper quadrants were more affected than the lower ones, and, between the lower ones, the temporal quadrant was more affected as compared to the nasal one.

**Figure 5:**
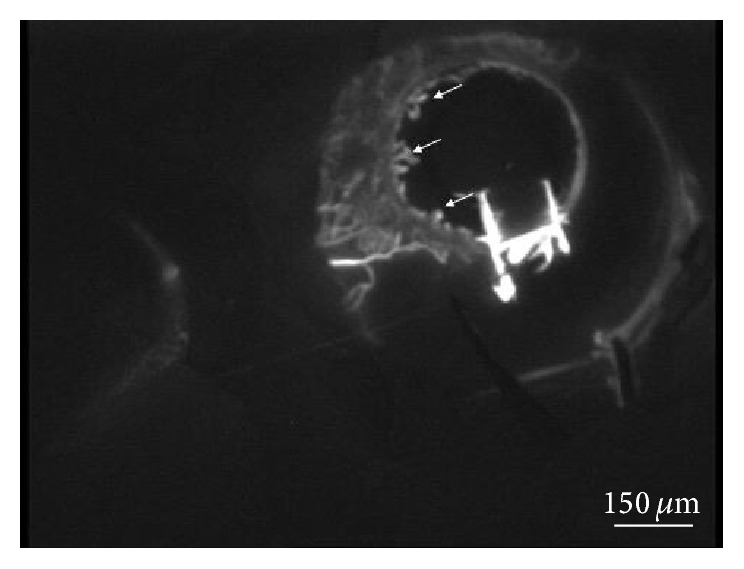
Retrograde filling furthest in Schlemm's canal was observed in the quadrants where the pathway was functioning (arrows).

Simultaneously a retrograde filling, of glomerular-shaped structures, furthest in Schlemm's canal occurred in the quadrants where the pathway was functioning (Figure 5). We hypothesize that these structures might be located in the iris rather than in the trabecula, since they did not show any leakage of dye in the anterior chamber in the late phases of the exam. Finally the aqueous veins, easily recognizable for their larger caliber, appeared. From the dye injection, the outflow structures remained visible for a period of 2.5 hours.

## 4. Discussion

Herein we showed that injection of a mixture composed of ICG and viscoelastic solution into the SC allows clear visualization of the portions of the conventional outflow pathway in patients affected by POAG. In addition, a retrograde filling of structures presumably located at the iris level was also noted. Various techniques have been employed in the past to visualize the outflow pathway [7–11]. In these previous studies, fluorescein was the most utilized dye. Disadvantages of fluorescein, due to its small molecular weight, include the need of large volumes and high concentration when injected directly into the AC, delayed filling of the episcleral venous network, and leakage from scleral vessels. More recently, Grieshaber et al. [7] injected fluorescein dye directly into Schlemm's canal using a flexible microcatheter during the canaloplasty. However, visualization of the intrascleral (deep) channel network and of the uveoscleral pathway was hindered by the immediate dye diffusion in the AC and perilimbal tissue and by the staining of the corneal endothelium and the anterior lens capsule. Furthermore, fluorescein passed the trabecular meshwork in the direction opposite that of the physiologic pathway, thus possibly not reflecting true permeability [14]. Therefore, the obtained images showed exclusively the episcleral veins [15].

Imaging of the SC is particularly complex due to the small size of the outflow structures and, even more, by the location which is several hundred microns beneath the sclera. High frequency ultrasound (UBM 50 MHz), OCT, and, more recently, SD OCT systems are the noninvasive procedures utilized to visualize the SC and the TM [8–11]. The weakness of these methods is due to the small size of the aqueous humor outflow structures and, even more, to the acquired images processing that further alters their size [9, 16]. Lastly, SD OCT presents static images, in which small vessels, differences among vessels, narrowing, and partial blockage of flow are not always evidenced or noticeable [17].

Conversely, ICG plus viscoelastic solution used in the present study allowed a dynamic examination of the aqueous humor conventional outflow pathway. An important finding was the visualization of the nonperfused portions of the outflow pathway. These* in vivo* results confirm previous* in vitro* histopathologic changes; in fact, Teng and coworkers [18], about 60 years ago, found in POAG eyes a degeneration of collagen and elastic fibers in the wall of the anterior chamber angle. The degeneration histologically began in the external trabecular region and spreads along these fibers internally or externally to Schlemm's canal, to the collector channels, and sometimes to the intrascleral plexus. The increase of this degenerative process was responsible for the subsequent drainage channels block. The same research group, two years later, identified other important histopathologic variations: increased collapse of SC and narrowing of collector channels [19]. These modifications identify SC and CCs as important anatomic structures responsible, together with TM, for the resistance to aqueous outflow in POAG eyes [20].

In our series, the filling of the CCs was somewhat independent of the patency of the SC since CCs might not be visualized in 4 quadrants despite perfusion of the SC over 360 degrees. In addition, the CCs closer to the site of injection did not necessarily fill up earlier than the farther ones. Actually, the filling seemed to be influenced by the resistance encountered during the dye progression within the lumen of the canal. In fact, after increasing the volume/pressure of infusion, visualization of some of the CCs, initially not perfused, occurred. Both of these findings further confirm that CCs themselves may represent a critical site in the outflow pathway [18–20]. Up to 50% of the resistance to aqueous humor flow occurs in the aqueous outflow pathway distal to TM, namely, at the level of SC and the CCs that join Schlemm's canal to intrascleral and episcleral vessels [21]. In keeping with this observation, we noted that the only noticeable aqueous veins were located in correspondence of the quadrant in which both the SC and the collectors were patent. The upper quadrants were more affected that the lower ones, and, between these, the temporal quadrant was more affected than the nasal one. It may be influenced by the AV distribution, which is not symmetrical. They are most commonly present in the inferior nasal quadrant, less in the inferior temporal quadrant, and more less in the upper quadrant [22].

This may suggest that shunt or collateral vessels bypassing blockage of the pathway at this level do not exist. In our glaucomatous patients, ICG plus viscoelastic solution canalography makes the visualization of the resistance site beyond TM possible. This could theoretically manage the surgical approach in a more rational way, intervening as close as possible to the site of impaired drainage, with the more appropriate choice. Thus, a customized glaucoma surgery, in keeping with the site of obstruction, might be considered to enhance the chances of success.

Another intriguing finding was the active retrograde filling of glomerular-shaped structures, furthest in the SC, observed in the quadrants where the pathway was functioning. We hypothesize that these structures might be located in the iris rather than in the trabecula, since they did not show any leakage of dye in the anterior chamber in the late phases of the exam. It is unlikely that they represent new vessels, as they were not visualized on ophthalmoscopy and the patients examined did not have a history positive for ischemic retinal diseases; moreover, no leakage from them at any time of the exam occurred. Contrarily, they could be lymphatic new vessels, whose development might be promoted by the increase in iris hydrostatic pressure secondary to POAG [23].

In conclusion, this study shows that the use of ICG coupled with viscoelastic solution, injected through the SC, is a useful tool to detect site of resistance in the outflow pathway. Research is ongoing to find a dye formulation that might be topically applied to visualize conventional AH outflow pathway in a noninvasive fashion.
